# Clinical Profile of Dengue Seropositive Infection From a Tertiary Care Hospital Situated in Mysuru, South India

**DOI:** 10.7759/cureus.65175

**Published:** 2024-07-23

**Authors:** Tejashree A, Chinchana E S, Satyasai Badveti, Krishna Karthik M VS, Vinay Kumar, Veerabhadra Swamy G S

**Affiliations:** 1 Microbiology, JSS (Jagadguru Sri Shivarathreeshwara) Medical College and Hospital, JSS Academy of Higher Education, Mysuru, IND; 2 Microbiology, Gayatri Vidya Parishad Institute of Healthcare and Medical Technology, Visakhapatnam, IND

**Keywords:** polymerase chain reaction (pcr), dengue haemorrhagic fever (dhf), dengue fever (df), reverse transcriptase polymerase chain reaction (rt-pcr), dengue shock syndrome (dss), dengue virus (denv), non-structural protein 1 (ns1), immunoglobulin m (igm)

## Abstract

Introduction

Dengue, a viral infection transmitted by *Aedes* mosquitoes, has become a significant global health concern. Its incidence has surged dramatically over the past decades, with severe cases potentially leading to life-threatening conditions such as dengue hemorrhagic fever (DHF) and dengue shock syndrome (DSS). Despite its prevalence in tropical regions, including India, the clinical manifestations of dengue can vary widely, sometimes presenting atypically. Recent outbreaks, particularly in Northern India, underscore the urgency of understanding and managing this disease. This study focuses on the clinical and laboratory findings of hospitalized dengue fever patients from January 2022 to January 2023, aiming to provide insights for effective patient care and mortality reduction.

Methods

This was a prospective study at JSS (Jagadguru Sri Shivarathreeshwara) Medical College and Hospital, Mysuru, Karnataka, India (January 2022-January 2023). Blood samples from suspected dengue patients presenting acute febrile symptoms were collected. NS1 antigen and IgM antibody were detected using enzyme-linked immunosorbent assay (ELISA). Patients positive for dengue NS1 antigen and IgM antibodies were included in the study, excluding those with co-infections or comorbidities.

Results

A nine-month study at JSS Hospital (January 2022-January 2023) screened 1019 samples, identifying 316 dengue cases. Among these, 84.8% were dengue fever and 15.1% were DHF/DSS. Male predominance (60.1%) was noted, with peak incidence in the age groups of 11-20 years (29.11%) and 0-10 years (27.53%). Common symptoms included fever (98.1%), headache (32.91%), myalgia (40.87%), and vomiting (42.7%). Thrombocytopenia was found in 60.6% of cases. NS1 was detected in 56% of patients and IgM was positive in 20.8% of the patients. Comorbidities like type 2 diabetes mellitus (T2DM) (7.59%) and hypertension (7.27%) were observed. Among severe cases, 43.6% had platelet counts <1 lakh/cumm, and 27.5% required intravenous fluids. Seven deaths occurred, primarily in patients with comorbidities and severe dengue.

Discussion and conclusion

High dengue seropositivity among males (60.12%) compared to females (39.87%) was noted, possibly due to varied exposures. Patients aged 11-20 years had the highest dengue infection, with a peak in admissions during the rainy season. Thrombocytopenia (60.6%) and comorbidities like T2DM and HTN were common, with seven fatalities linked to severe dengue and comorbidities, emphasizing the need for early recognition and management to reduce mortality.

## Introduction

Dengue is a viral infection induced by the dengue virus (DENV) and is typically transmitted to humans through the bites of infected mosquitoes [1]. The DENV belongs to the genus *Flavivirus* (renamed Orthoflavivirus in 2023) within the *Flaviviridae* family. The DENV has four distinct serotypes, namely DENV‐1, DENV‐2, DENV‐3, and DENV‐4 [2]. Over the past few years, dengue has emerged as a significant and pressing issue in global public health [3]. Dengue fever was initially reported in India in 1956, with the first instance occurring in Vellore. Subsequently, the first case of dengue hemorrhagic fever (DHF) in India was documented in Calcutta in 1963 [4].

The occurrence of dengue has surged by a staggering 30-fold over the past five decades. Presently, it is estimated that there are approximately 50-100 million dengue infections each year across more than 100 endemic countries, putting nearly half of the global population at risk [5]. As of October 2, 2023, the European Centre for Disease Prevention and Control (ECDC) has reported over 4.2 million cases of dengue and more than 3,000 dengue-related fatalities across 79 countries and territories worldwide [6]. About half of the global population is currently exposed to the risk of dengue, with an estimated range of 100-400 million infections reported annually. Dengue is prevalent in tropical and sub-tropical climates across the world, primarily affecting urban and semi-urban regions. In India, the yearly incidence of dengue is estimated to range from 7.5 to 32.5 million cases [5]

Dengue infections exhibit a spectrum of severity, spanning from mild, self-limiting flu-like symptoms to severe conditions such as DHF and dengue shock syndrome (DSS). If not promptly treated, DHF and DSS can be life-threatening, with mortality rates reaching as high as 20% [3]. In the past two decades, there has been a notable and substantial worldwide rise in the incidence of dengue fever, DHF, and DSS, along with their respective epidemics. The Southeast Asia region has witnessed an increase in the number of cases in the last three to five years. Within India, there is an observable rise in the proportion of dengue cases with severe manifestations. Dengue epidemics in India follow a cyclical pattern, occurring more frequently and extending to rural areas. Furthermore, all serotypes of DENV are circulating within the community [7].

The clinical manifestations of dengue are not always clearly defined, and in addition to the typical symptoms, there has been an increasing frequency of reports regarding rare and atypical presentations including cardiac, GI, neurological, and renal manifestations [5]. Some of these non-classical presentations, which do not fit neatly into the World Health Organization (WHO) definitions, could potentially be serious and contribute to higher morbidity and mortality associated with the disease. Many of these unusual manifestations may go unnoticed and unreported due to a lack of awareness among primary care physicians [3].

The most recent dengue outbreak occurred in northern India in 2022, specifically during the monsoon and post-monsoon seasons [8]. As per information from the WHO, untreated cases of dengue fever had a reported mortality rate of up to 20%. However, for patients who were hospitalized, the mortality rate was less than 1% [5].

Understanding the precise clinical characteristics is of paramount importance for effective patient management and, ultimately, for saving lives [9]. The current study aims to outline the notable clinical and laboratory observations based on serologically confirmed cases of hospitalized dengue fever patients.

## Materials and methods

This was a prospective study conducted in the Department of Microbiology, JSS (Jagadguru Sri Shivarathreeshwara) Medical College and Hospital, Mysuru, Karnataka, India, from January 2022 to January 2023. The study was approved by the Institutional Ethical Committee of JSS Medical College and Hospital (approval number: JSS/MC/PG/32).

All patients who presented with fever and were found positive for dengue NS1 antigen and IgM antibodies capture enzyme-linked immunosorbent assay (MAC-ELISA) were admitted to the ward and included in the present study. Patients with other co-infections like malaria and typhoid or with any other co-morbid diseases were excluded from the study.

A total of 316 blood (serum) samples were collected from the suspected dengue patients who were admitted to medical wards and who appeared to have an acute febrile illness with myalgia, arthralgia, headache, retro-orbital pain, abdominal pain, nausea and vomiting, bleeding, hypotension, or thrombocytopaenia, and were processed for the detection of NS1 antigen and IgM antibody using MAC-ELISA and NS1 ELISA according to manufacturer’s protocol.

A detailed history as well as the results of general and systemic clinical examination were recorded. Haematological profiles and biochemical investigations were done at the time of admission and were followed by daily investigations as required until discharge. Demographic data and details of clinical history and laboratory investigations were collected.

Statistical analysis

All statistical analyses were conducted using IBM SPSS Statistics for Windows, Version 26.0 (Released 2019; IBM Corp., Armonk, New York, United States).

## Results

During the study period of 12 months, 1019 dengue-suspected samples were screened for dengue IgM antibody and NS1 antigen by MAC-ELISA and enzyme-linked fluorescence assay (ELFA). Of the 1019 samples, 316 tested positive for either NS1 antigen, IgM antibody, or both. Of the 316 serologically confirmed cases, 268 (84.8%) conformed to dengue fever and 48 (15.1%) to DHF/DSS as per WHO case definition. Among the 48 cases of DHF/DSS, 31 patients presented with DHF and the remaining eight presented with DSS. Male predominance was observed at about 60.1%. The highest number of patients testing positive for dengue antigen (Ag) or antibody (Ab) belonged to the age groups of 11-20 years (n=92, 29.11%) and 0-10 years (n=87; 27.53%), followed by 21-30 years (n=66, 20.88%), 31-40 years (n=28, 8.88%), 41-50 years (n=19, 6.01%), and 24 (7.59%) patients were above 50 years of age (Table 1). The highest number of patients who were positive for DHF/DSS were in the age group of 0-15 years (n=26, 54.16 %) followed by 16-25 years (n=5, 10.41%), 36-45 years (n=5, 10.41%), and 26-35 years (n=1, 2.08%) (Table 1).

**Table 1:** Distribution of the study population by age and sex (N=316)

Characteristics		Frequency	Percentage
Age (in years)	0-10	87	27.53%
	11-20	92	29.11%
	21-30	66	20.88%
	31-40	28	8.88%
	41-50	19	6.01%
	More than 50	24	7.59%
Sex	Male	190	60.1%
	Female	126	3.98%

The most common symptoms were fever (n=310, 98.1%), followed by headache (n=104, 32.91%), myalgia (n=126, 40.87%), vomiting (n=135, 42.7%), abdominal pain (n=107, 33.54%), loose stool (n=98, 31.01%), breathlessness (n=47, 14.87%), bleeding manifestation (63, 19.93%), and petechiae (n=67, 21.20%) (Table 2).

**Table 2:** Clinical presentation of dengue positive cases (N=316)

Clinical manifestation	Frequency	Percentage
Fever	310	98.1%
Vomiting	135	42.7%
Myalgia	126	40%%
Abdominal pain	106	33.54%
Headache	104	32.91%
Loose stool	98	31.01%
Petechiae	67	21.20%
Bleeding manifestation	63	19.93%
Breathlessness	47	14.87%

Fever was the most common presenting symptom. Haematological investigations revealed thrombocytopenia in 160 (60.6%) cases. In our study, most patients (n=178, 56.32%) were found positive for NS1 antigen followed by IgM antibody (n=66, 20.88%) than for both NS1 and IgM (n=74, 23.41%). A total of 267 (84.4%) patients had a history of ≤5 days of fever, while 49 (15.5%) reported after >5 days of fever. Within five days of fever, 162 (51.2%) patients were positive for NS1, followed by NS1 and IgM (n=48, 15.1%) patients, IgM (n=57, 18.0%), and the remaining 49 (15.5%) patients were positive for NS1 and IgM after five days of fever, as more number of patients presented within five days of fever (Table 3).

**Table 3:** Correlation between number of days of fever and serological tests

Serological test	Number of positives (fever)		Total
	≤5 days	>5 days	
NS1	162	16	178
IgM	48	18	66
NS1 + IgM	57	15	72
Total	267	49	316

Many patients presented with comorbid conditions and coinfections such as pneumonia in six (1.89%) patients, followed by T2DM in 24 patients (7.59%), hypertension in 23 patients (7.27%), hepatitis in seven patients (2.21%), chronic kidney disease in seven (2.21%) patients, seizures in 12 (3.79%) patients, acute kidney injury in six (1.81%) patients, heart disease in 13 (4.11%) patients, depression in two (0.63%) patients, hypothyroidism in seven (2.21%) patients, acute gastroenteritis in three (0.94%) patients, chronic obstructive pulmonary disease in one patient (0.31%), chronic myeloid leukaemia in two patients (0.63%). Predominant system involvement was haematological involvement followed by hepatic, GI, renal, respiratory, and CNS involvement, and shock (Table 4).

**Table 4:** Systems involvement in dengue patients (N=316)

Systems	Frequency	Percentage
Haematological	160	50.6%
Hepatic	93	29.4%
Gastrointestinal	89	28.1%
Renal	57	18%
Respiratory	45	14.2%
Shock	23	7.27%
CNS	13	4.11%

Out of 316 patients, 138 (43.6%) had a platelet count of less than one lakh /cumm. Among them, 82 patients (25.9%) had a count of less than 50,000 / cumm, and 14 patients (4.4%) had a count of less than 10,000/ cumm. Among the 316 patients, 87 (27.5%) patients needed intravenous fluid, of which the majority (n=59, 67.81%) were severe dengue cases. Single donor platelet was given in 26 (8.22%) cases of which, most (n=21, 80.76%) were severe dengue cases. Packed red blood cells were given for nine severe dengue cases. Fresh frozen plasma was needed in eight cases and all of them were severe dengue cases (Table 5).

**Table 5:** Transfusion pattern (N=316)

Blood products	Frequency	Percentage
Single donor platelet	26	8.22%
Packed red blood cell	9	2.8%
Fresh frozen plasma	8	2.5%

Out of 316 patients, death occurred in seven (2.2%) patients who had different comorbidities like T2DM, hepatitis, hypertension, depression, myocarditis, and seizures. All the patients presented with DSS and DHF. The risk of dying was significantly increased in patients who had severe dengue infection along with comorbidities and aged more than 50 years.

## Discussion

The DENV, a spherical, enveloped RNA virus, belongs to the *Flaviviridae* family, specifically the *Flavivirus* genus. Its transmission primarily relies on two mosquito species: *Aedes aegypti*, widespread globally, and *Aedes albopictus*, prevalent in the United States, Asia, Latin America, and the Caribbean [8]. Dengue infection arises from four antigenically distinct viral serotypes: DENV-1, DENV-2, DENV-3, and DENV-4. Contracting an infection from one serotype confers lifelong immunity to that particular serotype, yet provides only partial and temporary safeguarding against potential subsequent infections from the other three serotypes [5]. Successive infections with various DENV serotypes elevate the risk of developing DHF. This phenomenon is attributed to a process called antibody-dependent enhancement (ADE). ADE occurs when non-neutralizing or sub-neutralizing levels of antibodies from a previous dengue infection facilitate the entry of a different DENV serotype into host cells, exacerbating the disease severity. Noteworthy is the fact that around 90% of DHF cases occur among children under 15 years of age. Presently, targeted treatments for DENV infections are lacking. Consequently, controlling the vector, primarily mosquitoes, remains the sole approach for preventing dengue transmission [8].

In the current study, the dengue positivity was high in males (n=190, 60.12%) in comparison with females (n=126, 39.87%). This is in agreement with other studies by Kaur et al. [8], Imam and Prashanth [10], Kauser et al. [11], Singh et al. [12], and Gupta and Bansal [13]. This variation in gender preponderance could be because of different social, occupational, and behavioural practices, which exposed them to anthropophilic *Aedes* mosquitoes that bite throughout the day.

In the current study, patients in the age group of 11-20 had the highest rate of dengue seropositivity of about 92 (29.11%), followed by 87 (27.53%) aged 0-10 years, and 66 (20.88%) aged 21-30 years. Adults aged 15-50 years were shown to be the most impacted age group (n=171, 54%), followed by children belonging to the age group of 0-14 years (n=145, 60%). Our findings were similar to the study conducted by Kauser et al. as the majority of those who suffered in their study were aged 20-30 years [11].

In a present study, out of 316, 268 (84.81%) were diagnosed with dengue fever and 48 (15.1%) with DSS and DHF. Our findings were consistent with a study by Singh et al., in which there were 178 (82.4%) cases of dengue fever, 31 (14.35%) of DHF, and seven (3.24%) of DSS [14], and with the study by Daniel et al., in which 166 (66.4%) conformed to dengue fever and 84 (33.6%) to DHF/DSS [9].

In this study, a notable trend emerged revealing a peak in-patient admission during the rainy season, specifically from June to October. This period aligns with the flourishing environment for the *A. aegypti* vector, known for its prominence in transmitting dengue fever, underscoring the correlation between environmental conditions and disease prevalence. This correlates with another study conducted by Kaur et al. [8], Imam and Prashanth [10], and Kauser et al. [11]. Mishra et al.'s study showed a distinct pattern of hospital admissions, predominantly occurring during the confluence of rainy and winter seasons, spanning from July to November [4].

The current study revealed fever was the most common symptom (n=310, 98.1%), followed by headache, myalgia, vomiting, abdominal pain, loose stool, breathlessness, bleeding manifestation, and petechiae. This correlates with another study conducted by Vijay et al., in which all cases were diagnosed with fever (100%), myalgia, headache, as well as joint pain, which were observed in higher frequencies [15]. Another study by Kaur et al. revealed fever as the most common presenting symptom (99.76%) [8]. It is crucial to note that various infections presenting with fever and gastrointestinal symptoms such as typhoid, leptospirosis, and enteroviral infections are prevalent in India. This diversity of infectious agents can sometimes contribute to a diagnostic delay in identifying dengue cases [8]. 

In our study, haematological investigations revealed thrombocytopenia in 160 (60.6%) cases. This correlates with the other studies conducted by Ageep et al. (88% of cases) [16] and Mittal et al. (92.6% of cases) [17].

Patients who were seropositive for dengue infection in the current study had different types of comorbidities like hepatitis, pneumonia, TSDM, hypertension, chronic kidney disease, seizures, myocarditis, and depression. Among the 316 patients, the majority (about 10%) had T2DM, hypertension, pneumonia seizures, and myocarditis. Our findings were similar to the study conducted by Kaur et al. in which acute respiratory distress syndrome was observed in 1.67% of cases, while myocarditis and seizures were each documented in 1.44% of cases [8]. Hemarthrosis and encephalopathy were both rare, with an occurrence of 0.24%.

In our study, the majority of the patients (56.32%) were found positive for NS1 antigen followed by IgM antibody (20.88%), and then for both NS1 and IgM (23.41%). A total of 267 (84.4%) patients had a history of ≤5 days of fever, while 49 (15.5%) reported after >5 days of fever. Within five days of fever, 162 patients were positive for NS1, followed by NS1 and IgM positivity in 48 patients, IgM positivity in 57 patients, and the remaining 49 patients were positive for NS1 and IgM after five days of fever, as a greater number of patients presented within five days of fever. This is similar to another study conducted by Damodar et al. [1]. In their case, the majority of about 40% of cases were positive for NS1 and 7% were positive for Igm. Out of 285 patients, 139 (48.8%) patients reported to the hospital within five days of fever, while 146 (51.2%) reported after >5 days of fever. Eighty-three (29%) cases were positive by at least one of the following tests: IgM ELISA, IgG ELISA, NS1 ELISA; 55 (65%) patients had a history of ≤5 days of fever, and 29 (35%) patients had a history of >5 days fever [1].

Detecting dengue cases early using the NS1 assay significantly aids in diagnosing and confirming instances of the disease. Specifically, the NS1 antigen detection method proves particularly effective within the initial five-day window of illness. It demonstrates heightened sensitivity in identifying primary dengue infections compared to secondary ones. NS1 helps differentiate primary and secondary dengue infections by its prolonged presence in primary infections compared to secondary infections. In primary infections, NS1 Ag remains detectable in the blood for a longer period, while in secondary infections, it is cleared more rapidly due to the presence of cross-reactive Abs. This diagnostic approach serves as a valuable tool in distinguishing and confirming dengue cases, especially during the early phase of the illness and in discerning between primary and secondary infections.

Out of 316 patients in the current study, 138 (43.6%) had a platelet count of less than one lakh /cumm. Among them, 82 patients (25.9%) had a count of less than 50,000/cumm and 14 patients (4.4%) had a count of less than 10,000/cumm. This is similar to the study by Daniel et al., which revealed that 225 patients, constituting 90% of the total cases, exhibited a platelet count falling below 100,000/cumm [8]. Within this subgroup, a significant proportion, specifically 48%, representing 108 patients, displayed an even more pronounced reduction in platelet count, registering below 50,000/cumm. Of notable concern, a subset of 8.4%, comprising 19 patients, experienced an acute decline in platelet count to levels below 10,000/cumm.

Of the 316 patients in the current study, 43 (27.5%) needed intravenous fluid, single donor platelet was given in 26 (8.22%) cases, and PRBC was given for nine (2.8%) severe dengue cases. Fresh frozen plasma was needed in eight (2.5) cases and all of them were severe dengue cases. This correlates with the studies by Padyana et al. [18] and Kaur et al. [8], in which an average of two units of single donor platelet transfusion requirement per patient was observed and transfusions were needed in the form of random donor platelets and single donor platelets in 139 (33.25%) and 115 (27.51%) patients, respectively. However, in 10 (2.39%) patients, packed cells were transfused.

In our study, death occurred in seven (2.2%) patients, and mortality was significantly associated with different comorbidities like T2DM, hepatitis, hypertension, depression, myocarditis, and seizures. All the patients presented with DSS and DHF. The risk of dying was significantly increased in patients who had severe dengue infection along with comorbidities, age group more than 50 years, and a platelet count less than 50000. This correlates with studies by Kashinkunti et al. [19], Jain et al. [20], Bhushan and Kumar [21], Daniel et al. [9], Avarbeel et al. [22], in which deaths were 3%,6%, 1.7%, 4.4%, 3.2%, and 2%, respectively.

Limitations

The study was conducted in a single tertiary care hospital in Mysuru, South India, which may limit the generalizability of the findings to other regions. Serotyping and genotyping of the positive samples were not performed, and secondary dengue infections were also not investigated or elicited. Additionally, the duration was limited to 12 months, which may not have captured seasonal variations and long-term trends in dengue infection.

## Conclusions

Dengue is a serious global health issue. Recognizing unusual dengue symptoms is vital for early diagnosis and preventing severe complications. Timely diagnosis and treatment significantly lower death rates. Our study, conducted in South India, documented typical and unusual dengue symptoms. We found varied symptoms and risks such as bleeding tendencies and low platelet count, leading to higher mortality. Better transport, swift treatment, and WHO-guided care can further reduce deaths. Monitoring symptoms and lab results is crucial for dengue management. Though vaccines are in development, more research is needed for a better understanding of dengue.
